# Chemical Profile and Anti-inflammatory Activity of Total Flavonoids from *Glycyrrhiza Uralensis* Fisch

**Published:** 2018

**Authors:** Lei Yin, Enshuang Guan, Yuanbin Zhang, Zhiheng Shu, Bing Wang, Xiuli Wu, Jing Chen, Jingxia Liu, Xueyan Fu, Weihong Sun, Meifeng Liu

**Affiliations:** a *School of Pharmacy, Ningxia Medical University, Yinchuan 750004, China. *; b *Ningxia Engineering and Technology Research Center for Modernization of Hui Medicine, Yinchuan 750004, China.*; c *School of traditional Chinese medicine, Ningxia Medical University, Yinchuan 750004, China. *; d *Key Laboratory of Hui Ethnic Medicine Modernization, Ministry of Education * *(* *Ningxia Medical University, Yinchuan 750004, China.*; e *Depterment of General Hospital of Ningxia Medical University, Yinchuan 750004, China. *; f *School of Chemistry and Chemical Engineering, Guangdong Provincial Key Lab for Green Chemical Product Technology, South China University of Technology, Guangzhou 510640, China.*; 1 L.Y, E.G, Y.Z and Z.S. equally contributed to this Article.

**Keywords:** Anti-inflammatory, Glycyrrhiza uralensis Fisch, Total flavonoids, HPLC, ELISA

## Abstract

*Glycyrrhiza uralensis* Fisch. (*G. uralensis*) is one of the most widely used herbal medicines. This study was designed to enrich total flavonoids (TFF) from *G. uralensis*. The chemical profile of TFF was identified by HPLC and colorimetric assay. The TFF mainly contained liquiritin apioside, liquiritin, isoliquiritin apioside, liquiritigenin and isoliquiritigenin without glycyrrhizic acid. To study the anti-inflammatory activity of TFF, the DMB-induced ear vasodilatation assay and carrageenan-induced rat paw edema model have been utilized. Treatment with TFF showed significant anti-inflammatory activities in the two models. The two *in-vivo* edema assays demonstrated that the TFF possesses significant dose-dependent anti-inflammatory activity, similar to that of indomethacin at a dose of 500 mg/kg. In rat paws with carrageenan, treatment with TFF (500 and 250 mg/kg) markedly inhibited the expression of IL-1β and iNOS. TFF at all doses noticeably decreased levels of NO and MDA at the site of inflammation, while only i.g. TFF at a dose of 500 mg/kg significantly decreased TNF-α levels in the carrageenan-injected paws. In addition, an increase in SOD activity was induced by TFF at all doses. These results revealed that TFF exhibited significant anti-inflammatory activity in acute inflammatory models.

## Introduction

*G. uralensis* is a widely used herbal medicine in Asia and southern Europe. It is one of the most widely used herbal medicines for the treatment of cough, spasms, pain, inflammation and low immunity (1, 2). Current research about *G. uralensis* is mainly concentrated on the pharmacological functions of polysaccharides, saponins and flavonoids*.* Various pharmacological activities of *G. uralensis* and its main constituents (polysaccharides, glycyrrhizic acid and glycyrrhetinic acid) have been extensively studied, including anti-inflammatory (3-7), anti-cancer (8, 9) and immunomodulatory (10, 11) effects in animals and cells. Flavonoids are secondary plant metabolites. They exhibit various biological activities and are used in health-care, food and medicine. Some studies have demonstrated that total flavonoids from *G. uralensis* have anti-inflammatory properties in cells (12,13). The flavonoids isoliquiritigenin and licochalcone A have been shown to inhibit inflammation in different models (13-17). However, whether TFF processes anti-inflammatory activity *in-vivo* and the possible mechanisms explaining how TFF may inhibit the inflammatory response are unknown. 

 In this study, the anti-inflammatory activity of TFF was evaluated with the edema assay *in-vivo*. iNOs, NO, TNF-α, IL-1β, and MDA levels and SOD activities at the site of inflammation were assayed, accompanied by the phytochemical analysis by HPLC to identify the constituents of TFF. The aim of this research was to study the possible active ingredients in the anti-inflammatory effects of TFF, and to provide evidence for its traditional uses in fighting inflammatory disease. These studies will identify new active fractions or chemicals from *G. uralensis *that could be further exploited for use in daily life.

## Experimental

*Herbal preparation and extraction*

 The roots of *G. uralensis* were harvested from Yanchi, Ningxia, China in June. The plant sample was authenticated by Xinhui Zhang at the Pharmacognosy Department, college of Pharmacy, Ningxia Medical University, and a voucher specimen was deposited in the same unit (herbarium number: 2013612).

*G. uralensis* (3 kg) was extracted using ultrasonic techniques three times with 70% ethanol (mass ratio of solid to liquid was 1:8; 30 min). The extract was then combined and evaporated to dryness under reduced pressure. Deionized water was added to the crude extract to obtain sample solutions (15 L). The sample solutions were pumped at a flow rate of 2 BV/h, into the properly treated chromatography column pre-packed with AB-8 macroporous resin (4 L). After sample loading, deionized water was used to wash the column until the effluent appeared colorless in the first step, followed by elution with 8 BV of 70% (v/v) ethanol aqueous solution. Eluates were collected and evaporated under reduced pressure until no alcohol taste was present. The solution was then partitioned with ethyl acetate to yield an ethyl acetate fraction. The dry ethyl acetate fraction was dissolved in deionized water (3 L) and adsorbed on a polyamide resin (5 L). The flow rate was 2 BV/h. The column was then washed with deionized water until the effluent was colorless and then eluted with 70% (v/v) aqueous ethanol (8 BV). Eluates were collected and evaporated to dryness using a rotary evaporator to yield a total flavonoids fraction (TFF). 

*Estimation of the total flavonoids content*

 The total flavonoids content was determined using a potassium hydroxide colorimetric assay. First, 0.5 mL of diluted solution containing flavonoids and 0.5 mL of 10% (w/w) KOH were added to a volumetric flask (10 mL). The solution was then diluted with 70% ethanol to volume, and mixed. After 5 min at room temperature, the absorbance of the solution was measured at 337 nm using a UV spectrophotometer against the same mixture, without the sample as a blank. The total flavonoids content was expressed as liquiritin equivalents.

*HPLC analysis*

 HPLC analysis was performed with an Agilent 1100 HPLC system equipped with a diode array detector (DAD). A Welchrom-C18 column (4.6 × 250 mm, 5 μm) was used. Gradient elution was employed using solvent systems A (acetonitrile) and B (0.2% glacial acetic acid). The flow rate was 1.0 mL/min and column temperature was maintained at 25 °C. The detector wavelength was 310 nm. An aliquot of 10 μL of solution was injected for acquiring chromatograms. The gradient program is shown in Table 1.

*Drugs and chemicals*

The following drugs and chemicals were used: dimethylbenzene and ethanol were purchased from Damao Chemical Company (Tianjin, China). Carrageenan (Grade: BR; no: YY13755-25 g) was obtained from Shanghai Yuanye Biological Technology Co., Ltd. Indomethacin (no. A130602) was obtained from Shanxi Yunpeng Pharmaceutical Co., Ltd. The ELISA kit for rat TNF-α, IL-1β, NO, iNOS, SOD and MDA were obtained from the Beijing Cheng Lin Biological Technology Co. Ltd. (China). TTF was suspended in water with 0.5% w/v sodium carboxyl methyl cellulose (Na-CMC).

*Animal preparation*

ICR mice (18-22 g; License No: SYXK (NING) 2011-0001) and Sprague-Dawley rats (180-220 g; License No.: SCXK (NING) 2012-0001) were purchased from the Experimental Animal Center of Ningxia Medical University (Ningxia, China). They were maintained in standard laboratory cages, with moderate humidity (50% ± 5%), at a constant temperature (22 ± 1 °C) in a 12-h light-dark cycle room. All animals had free access to food and water during the experimental period. The experimental procedures were approved by our institutional animal research ethics committee.

*Toxicity study*

The acute toxicity study of TFF was performed according to Modern Pharmacology Experimental Methods (volume Ⅱ) (18) guidelines (2012). The TFF was suspended in water with 0.5% (w/v) sodium carboxyl methyl cellulose (Na-CMC); doses of 500, 1000, 2000 mg/kg body weight were orally administered to over-night-fasted, healthy mice (n = 8), and the animals were observed continuously for 24 h for mortality.

*Dimethylbenzene (DMB)-induced ear vasodilatation assay*

This was carried out according to previously described methods (19). Mice were randomly divided into five groups (n = 8): control group, an indomethacin (10 mg/kg) positive group and three TFF groups (500, 250 and 125 mg/kg). Treatment were administered via oral gavage. After 40 min, 30 μL of DMB was applied to both the inner and outer surfaces of the right ear; the left ear was considered as the control. Mice were sacrificed by cervical dislocation, and both ears were removed 30 min later. Biopsies of both ears were obtained with a punch (a diameter of 8 mm) and weighed. The difference in weight caused by the irritant was measured by subtracting the weight of the untreated left ear section from that of the treated right ear sections.

*Carrageenan-induced paw edema*

Paw edema was induced according to Winter *et al*. (20). The animals were divided into five groups of seven each. They were pretreated orally with either vehicle, indomethacin (10 mg/kg) or TFF (500, 250 and 125 mg/kg). After 40 min, edema was induced with the injection of 0.1 mL of 1% (w/v) freshly prepared carrageenan suspension in saline into the right hind paw of each rat. Inflammation was quantified by measuring the volume (mL) displaced by the paw using a plethysmometer (Beijing Zhongshidichuang Science and Technology Development Co., Ltd, model YLS-7C, China) at 0, 1, 2, 3, 4 and 5 h after carrageenan injection. The edema volume was expressed as the change in volume between each time point (1, 2, 3, 4 and 5 h) and at basal time (0 h).

At the end of the experiments, the animals were sacrificed and all right hind paws were dissected and stored at −80 °C. Skin tissue was homogenized in PBS (pH = 7.4). The homogenates were centrifuged at 2000 ×g for 20 min at 4 °C, and the supernatant was removed. Repeated freeze-thaw cycles were avoided. The concentration of inflammatory cytokines, inflammatory mediators and antioxidant factors (TNF-α, IL-1β, NO, iNOS, MDA and SOD) was measured by ELISA. Assays were performed according to the manufacturer’s instructions.

*Statistical analysis*

Results were analyzed using the statistical program SPSS Statistics, version 17.0. One-way ANOVA followed by Dunnett’s test or Dunnett’s T3 test were used for determining the statistically significant differences between experimental groups. Values of *P < 0.05 and **P < 0.01 were regarded as statistically significant.

## Results

**Figure 1:**
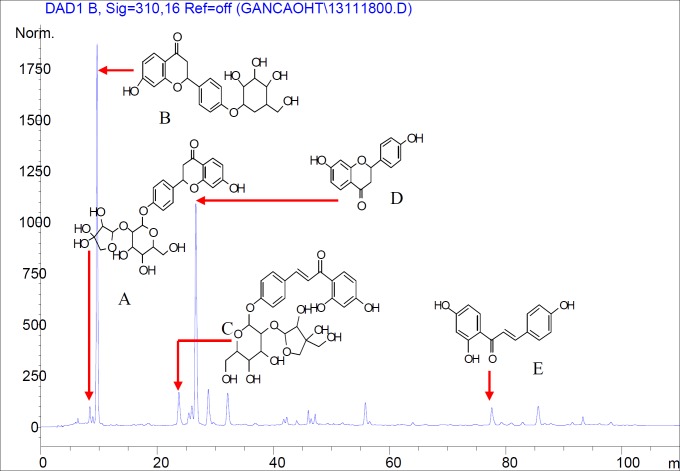
HPLC chromatogram of TFF (Major peaks were identified by comparison with standard compounds. Liquiritin apioside (A), liquiritin (B), Isoliquiritin apioside(C), liquiritigenin (D) and Isoliquiritigenin (E

The total flavonoids content expressed as liquiritin equivalents was 53.25% (y = 51.252 x - 0.0048, R^2^ = 0.9999). HPLC analysis indicated that the major peaks identified by comparing with standard compounds were liquiritin apioside (A), liquiritin (B), isoliquiritin apioside (C), liquiritigenin (D) and isoliquiritigenin (E) in TFF (Figure 1). Glycyrrhizic acid was not identified in TFF by HPLC analysis.

The TFF at various doses did not show any mortality, any symptoms of sickness or deleterious effects. The LD50 value by the oral route could not be determined as no lethality was observed for doses up to 2 g/kg in mice. This indicated a robust safety margin for TFF treatment.

**Figure 2:**
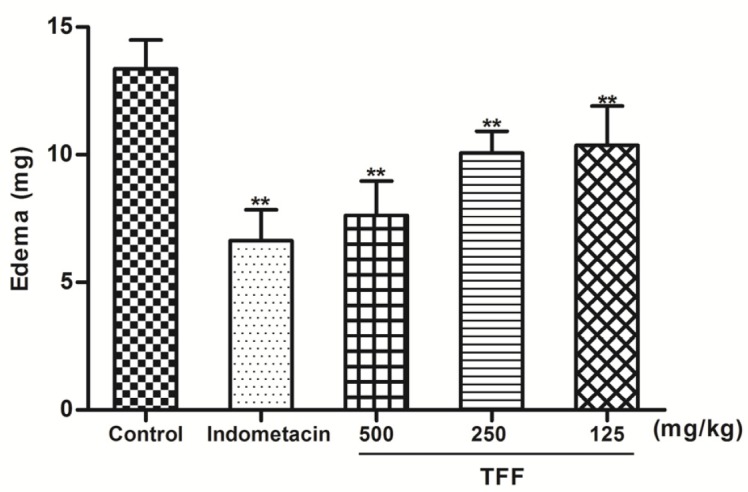
Effects of oral pretreatment with TFF and indomethacin (10 mg/kg) on DMB-induced ear vasodilatation. The results of ear edema are expressed as the mean ± S.D. Differences between the groups were determined by ANOVA followed by Dunnett’s test. *P < 0.05, **P < 0.01 compared to the control group

In the DMB -induced ear edema study, the inhibitory activity of oral TFF in mice is demonstrated in Figure 2. TFF at all doses and indomethacin (10 mg/kg) significantly (*P* < 0.01) inhibited ear edema formation in a dose-dependent manner. TFF at 500 mg/kg was as effective as indomethacin.

**Figure 3:**
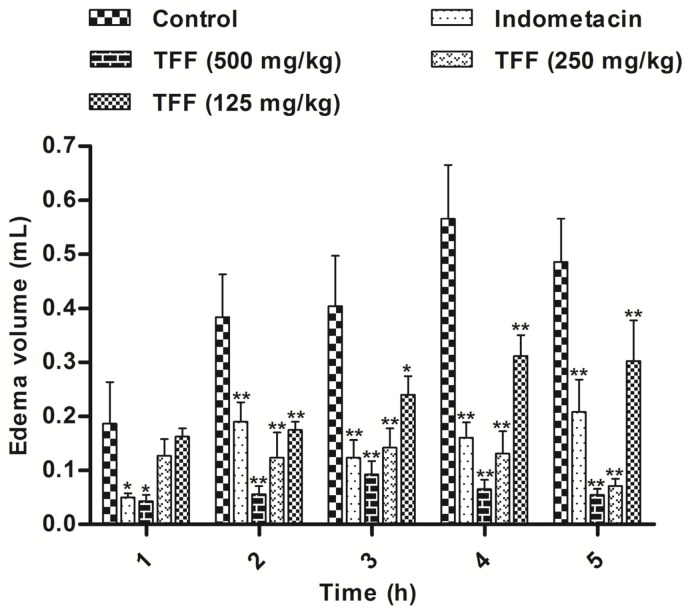
Anti-inflammatory activity of TFF and indomethacin (10 mg/kg) in carrageenan induced hind paw oedema. Each value represents the mean ± S.D. Differences from the control group were determined by ANOVA followed by Dunnett’s test or Dunnett’s T3 test. n = 8, *P < 0.05, **P < 0.01

TFF was assayed for anti-inflammatory activity using the inhibition of carrageenan-induced inflammation in mice. The results are shown in Figure 3. Treatment of animals with TFF at all doses (500, 250 and 125 mg/kg) and indomethacin (10 mg/kg), before injection of carrageenan significantly inhibited paw edema in rats at 1, 2, 3, 4 and 5 h compared to the controls. The TFF dose of 500 mg/kg demonstrated statistically significance inhibition at every hour with a maximum efficacy, similar to that of indomethacin (*P* < 0.01; *P* < 0.05). However, the edema at 1 h was only significantly inhibited by TFF at a dose of 500 mg/kg and by indomethacin (*P* < 0.05).

**Figure 4:**
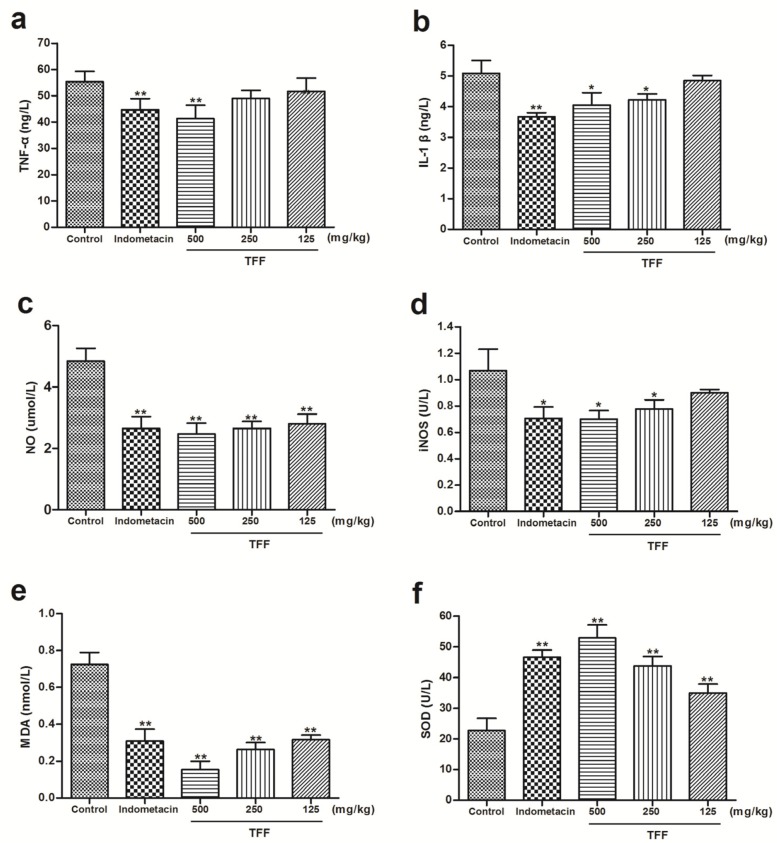
Effect of the different concentrations of TFF on the expression of TNF-α **(a)**, IL-1β (b), NO (c), iNOS (d), MDA (e) and SOD (f) in rat paws treated with carrageenan. Data are expressed as the means ±S.D. of six rats. Differences from the control group were determined by ANOVA followed by Dunnett’s test or Dunnett’s T3 test. (*P < 0.05, **P < 0.01; compared to the control group

As shown in Figure 4, pretreatment with TFF at 500 mg/kg significantly (*P* < 0.01) reduced the expression of TNF-α better than indomethacin (Figure 4a). Doses of 250 and 125 mg/kg did not show statistically inhibition. TFF at dose of 500 and 250 mg/kg significantly (*P* < 0.05) decreased the expression of IL-1β and iNOS (Figure 4b and d), but the difference did not reach statistical significance at 125 mg/kg. Compared to the control group, treatment of animals with TFF at all doses (500, 250 and 125 mg/kg) could decrease NO and MDA (*P* < 0.01, Figure 4c and e). In addition, the increase in SOD was attenuated by TFF at all doses (*P* < 0.01, Figure 4f). Indomethacin (10 mg kg^-1^) also inhibited TNF-α, IL-1β, iNOS, NO and MDA in rats paws compared to control, with the exception of SOD, which was increased by indomethacin (*P* < 0.01, *P* < 0.05, Figure 4).

## Discussion and Conclusion

Glycyrrhizic acid is the main ingredient in licorice saponins and the most important active ingredient in the licorice root, possessing a wide range of pharmacological and biological activities (21). Early studies from our laboratory found that glycyrrhizic acid cannot be removed from total flavonoids by a single separation method. This compound can compromise the evaluation of a total flavonoids fraction. In the current study, in order to avoid the interference of glycyrrhizic acid, three methods (liquid–liquid extraction, AB-8 macropriorous and the polyamide resin adsorption technique) were combined to enrich TFF and identified the purity of TFF by HPLC and colorimetric assay. The results showed no glycyrrhizic acid in TFF compared with a glycyrrhizic acid standard at 248 nm, the maximum absorption wavelength of glycyrrhizic acid. The content of TFF exceeds the requirements of animal experiments by more than 50%.

**Table 1:** The HPLC/DAD gradient program of TFF.

**Time（min）**	**A** **（%）**	**B** **（%）**
0	20	80
15	20	80
25	25	75
35	25	75
45	35	65
50	35	65
65	40	60
70	40	60
75	45	55
85	45	55
90	55	45
100	55	45
105	20	80
110	20	80

Inflammation is part of the complex biological response of vascular tissues to any external or internal harmful stimuli. It is involved in the pathophysiology of various diseases such as cancer, arthritis and vascular diseases (22). Inflammation always starts as an acute process characterized by marked vascular changes, including vasodilatation, increased permeability and decreased blood flow (23). In the current study, two animal models were evaluated. Ethylene-induced ear edema in mice is a preliminary and simple acute inflammation model for the evaluation of potential anti-inflammatory agents (24). Ear edema may involve inflammatory mediators such as histamine, kinin, fibrinolysin, phispholipase A2 and PLA2. These mediators induce edema by promoting vasodilation and increasing vascular permeability (25, 26). TFF at all doses (500, 250 and 125 mg/kg) was able to reduce inflammation in this model. These results suggest that TFF may interfere with the actions of inflammatory mediators to inhibit inflammation.

Carrageenan-induced paw oedema is largely used for screening both steroidal anti-inflammatory drugs and NSAIDs (27). The inflammatory response involves the sequential release of several mediators in three phases. The first phase (0-90 min) is characterized by the release of serotonin and histamine, the second phase (90-150 min) involves kinin and prostaglandins dominate the third phase (after 180 min) (28), as shown in Figure 3. Animals were treated with TFF at all doses (500, 250 and 125 mg/kg), but at 1 h, the edema was only significantly inhibited by TFF at a dose of 500 mg/kg and by indomethacin; the doses of 250 and 125 mg/kg may be too low to exert an inflammatory effect at 1 h, Treatment of animals with all doses inhibited paw edema in rats at 2, 3, 4 and 5 h. Many studies have shown that carrageenan injection also provokes the release of pro-inflammatory cytokines such as TNF-α and IL-1β (29,30). TNF-α is a mediator of Carr-induced inflammatory incapacitation, and is able to induce the further release of kinins and leukotrienes, which may have an important role in the maintenance of long-lasting nociceptive responses (31). IL-1β is also important in the regulation of the inflammatory response. IL-1β increases the expression of adhesion factors on endothelial cells to enable transmigration of leukocytes, and is associated with hyperalgesia (32). In this study, the results suggest that TFF possibly acts by inhibiting the release of TNF-α and IL-1β in the development of edema.

The L-arginine - NO pathway has been proposed to play an important role in the Carr-induced inflammatory response (33). Inducible nitric oxide synthase (iNOS), which generates numerous pro-inflammatory mediators, catalyzes the formation of nitric oxide (NO) from L-arginine (34). The expression of iNOS has been shown to be an important mediator of inflammation (35). In this study, there are significant decreases in iNOS activities with TFF treatment. The suppression of NO production is most likely due to decreases in iNOS activity.

Free radical generation at the site of inflammation is proposed to be the major cause of the tissue damage induced by many inflammatory disorders. It has been demonstrated that free radicals will be released after injection with Carr, and free radicals may attack the plasma membrane, resulting in the accumulation of MDA. MDA is believed to be one of the most important markers of free radical generation and the subsequent development of oxidative stress (36, 37). Tissue damage related to oxidative stress can be reversed via the SOD enzyme. The action of these parameters limits the cytotoxic effects of toxic free radicals (38). In this study, there are significant increases in SOD activity with TFF treatment. Furthermore, there are significant decreases in MDA levels. Oxidative stress may therefore be inhibited by TFF.

*G. uralensis* has been used as a traditional medicine to treat some diseases in China. In this study, total flavonoids were successfully obtained by liquid-liquid extraction, AB-8 macropriorous and polyamide resin adsorption with a higher content. The anti-inflammatory activities of the purified flavonoids were evaluated by two animal models, suggesting that total flavonoids of *G. uralensis* play a major role in anti-inflammatory actions. Flavonoids are major contributor to the anti-inflammatory effects of *G. uralensis*. These experimental results support the original hypothesis that TFF is a potent inhibitor of inflammation *in-vivo*. The knowledge gained from this study can be useful in further exploitation and application and provides evidence for the traditional uses of *G. uralensis* in treating inflammatory diseases in daily life. 
